# Highly pathogenic avian influenza (A/H5N1) virus outbreaks in Lesotho, May 2021

**DOI:** 10.1080/22221751.2022.2043729

**Published:** 2022-03-10

**Authors:** Mabusetsa R.J. Makalo, William G. Dundon, Tirumala B.K. Settypalli, Sneha Datta, Charles E. Lamien, Giovanni Cattoli, Moeketsi S. Phalatsi, Relebohile J. Lepheana, Mpaliseng Matlali, Relebohile G. Mahloane, Marosi Molomo, Palesa C. Mphaka

**Affiliations:** a Department of Livestock Services, Ministry of Agriculture and Food Security, Maseru, Lesotho; b Animal Production and Health Laboratory, Animal Production and Health Section, Joint FAO/IAEA Division, Department of Nuclear Sciences and Applications, International Atomic Energy Agency, Vienna, Austria; c Department of Animal Science, National University of Lesotho, Roma, Lesotho

**Keywords:** Highly pathogenic avian influenza, A/H5N1, Lesotho, full genome, phylogenetic analysis

## Abstract

In May 2021, Lesotho reported its first outbreak of highly pathogenic avian influenza (HPAI) to the OIE. Samples were collected from infected poultry and the virus was confirmed by molecular tests to be of the H5N1 subtype. Full genome sequencing and phylogenetic analysis revealed that the viruses belonged to clade 2.3.4.4b and showed high identity with A/H5N1 viruses identified in Nigeria and Senegal in early 2021. The identification of A/H5N1 HPAI in Lesotho has important implications for disease management and food security in the region.

Highly pathogenic avian influenza (HPAI) of the H5 subtype continues to be a threat to the global poultry industry in addition to having significant public health and food security implications [1]. Since its first identification in Guandong province, China in 1996, the Gs/Gd HPAI H5NX lineage has diversified into ten clades based on the hemagglutinin (HA) gene. Of these clades, strains from clade 2.2, 2.3.2.1c and 2.3.4.4b have been detected in avian populations, both wild and domestic, in Africa. The first detection of clade 2.3.4.4b was in wild birds in birds in Russia and China in 2016/2017 [2]. In 2021, there have been numerous reports of HPAIs outbreaks in South Africa in wild and domestic birds made to the World Organization for Animal Health (OIE) (OIE Reports 149492 and 150076) in addition to reports from Lesotho and Botswana of A/H5N1/H5NX outbreaks in poultry (Reports 150121 and 151536). According to another recent report by the Joint OIE-FAO Scientific network on animal influenza (OFFLU), the viruses identified in wild birds and poultry in South Africa and poultry in Botswana belong to clade 2.3.3.4b but the sequence data is not currently publicly available. This outbreak report from Lesotho includes the complete genetic characterization of the HPAI A/H5N1 involved.

On 28 May 2021, the death of 300 (10%) layer chickens in a farm of 3000 layers (Farm A) located in Ha Penapena, Maseru District, Lesotho was reported to the Central Veterinary Diagnostic Laboratory (CVDL) in Maseru, Lesotho. The farmer confirmed that the 3000 point-of-lay chickens had been recently sourced from a poultry supplier located in the Free State province of neighbouring South Africa. The chickens started dying shortly upon arrival from South Africa on 19 May 2021. On 28 May 2021 and 29 May 2021, Farm A was visited by a team from the Animal Health Division of the Department of Livestock Services. Dead chickens were observed while others were manifesting clinical symptoms including green diarrhoea, swollen face, coughing, cyanosis of comb and wattles, and paralysis. Twelve nasopharyngeal samples were collected and resuspended in 1 ml of sterile PBS, (see supplementary material Table S1). On 1 June 2021, a second farm (Farm B) located at Mahobong, Leribe district, reported high mortality (5%) in 2000 chickens which were also recently sourced from the same supplier in South Africa as Farm A. Nasopharyngeal swab samples (*n* = 13) were collected from dead chickens (see supplementary material Table S1).

On arrival at the CVDL, RNA was extracted from 200 µl of the swab suspensions collected from both farms using the RNeasy Mini kit (Qiagen, USA) and following the manufacturer’s instructions. Purified RNA (8 µl) was tested using the VetMAX™-Gold AIV Detection Kit (Thermo Fisher Scientific, USA) as recommended by the World Health Organization [3]. This duplex RT-qPCR detects both the Influenza A matrix (M) and nucleocapsid (NP) genes. Twelve samples from Farm A and ten samples from Farm B were confirmed positive for influenza A using this protocol. Next, the RNA from ten positive influenza A samples (five from each farm based on Ct values which ranged from 19 to 35) were further characterized using a second RT-qPCR protocol recommended by the WHO to determine whether the viral RNA was of the H5 subtype [3]. All samples were positive.

Eleven RNA samples (ten from Farm A and one from Farm B) were sent to the Animal Production and Health Laboratory (APHL) of the FAO/IAEA Joint Centre, Seibersdorf, Austria for confirmation and full genome sequencing analysis. The viral RNA was confirmed as belonging to A/H5N1 using a duplex RT-qPCR developed by the Freidrich-Loeffer-Institute, Insel-Reims, Germany [4].

The RNA from two samples, one from each farm, (i.e. 352.3 and 341.10) was amplified by RT–PCR according to the protocol of Zhou et al [5] and the full genome was sequenced (see supplementary material). The genomes of A/Chicken/Lesotho/352.3/2021 (GenBank OL477466-OL477474) and A/Chicken/Lesotho/341.10/2021 (GenBank OL477521-OL477528) were identical except for a single nucleotide difference in the PB2 gene resulting in a V575M change in the protein. The effect of this V575M mutation on polymerase activity in recombinant PR8 viruses has been tested but was shown not to increase replication efficiency [6].

Phylogenetic analysis using the complete HA gene revealed that both viruses belonged to subclade 2.3.4.4b and that they were very similar (98.93–99.93% nucleotide identity) to A/H5N1/H5N8 viruses identified in Nigeria and Senegal in 2021, respectively (Figure 1). The viruses from Nigeria were all identified in chickens while the virus from Senegal were identified in chickens and a great white pelican (*Pelecanus onocrotalus*) [7]. The cleavage site of the HA was PLREKRRKRGLF confirming that the viruses were highly pathogenic. In addition, the HA protein possessed QRG motif (positions 222–224 -H5 numbering) at the receptor-binding site indicating a preference for avian-like α2-3 receptors [8].

**Figure 1.**
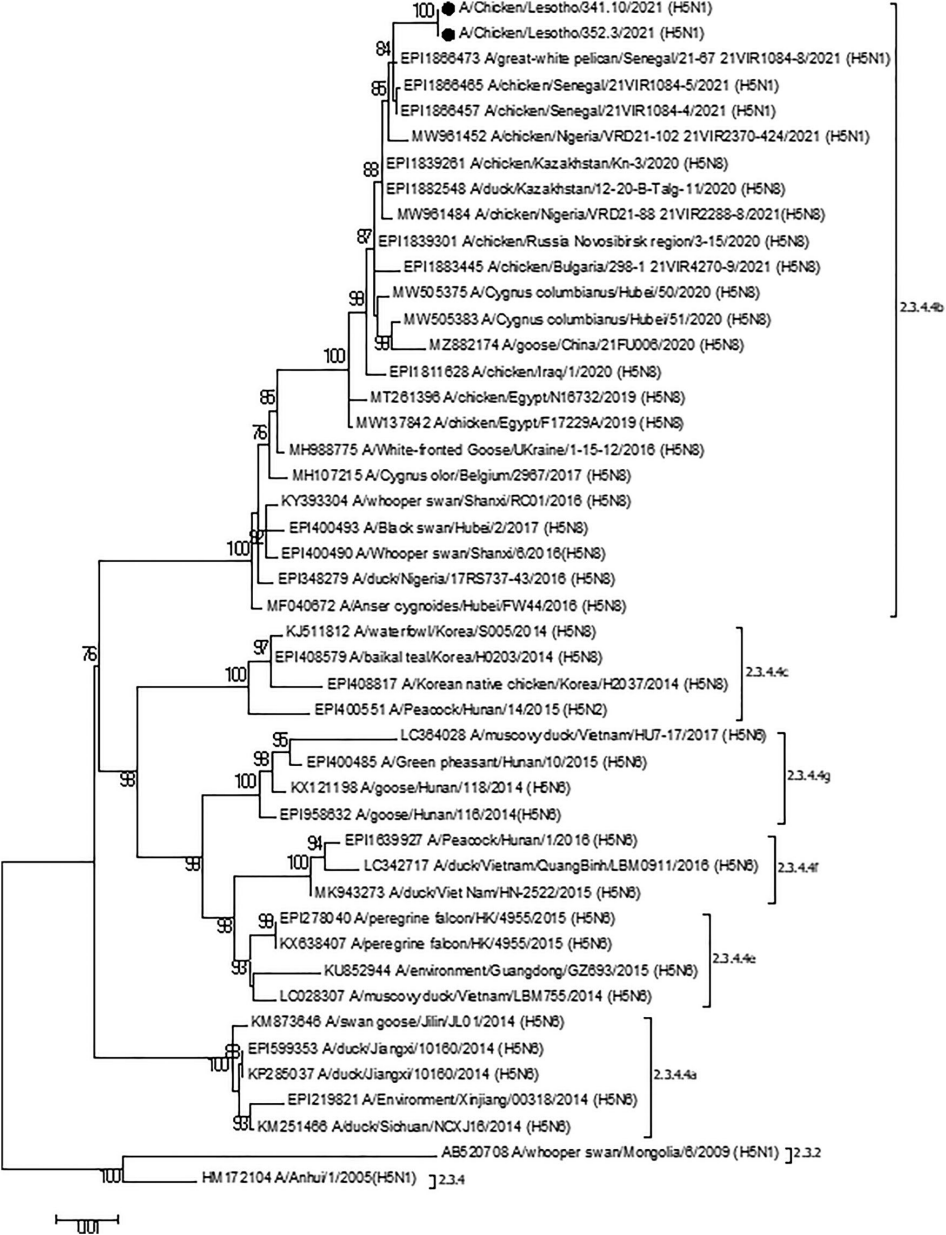
ML phylogenetic tree employing the Tamura-Nei model of nucleotide substitution with gamma distributed [with invariant sites (G + I)] rates among sites and 1000 bootstrap replications of the complete HA gene sequence (1704 bp) gene sequence from H5N1 HPAIVs collected in Mongolia combined with related sequences available in GenBank. The sequences from this study are shown by filled black circles. Different subclades of clade 2.3.4.4 are shown. Clade 2.3.2 and 2.3.4 are also indicated.

The open-reading frame (ORF) of the neuraminidase (NA) gene of both viruses was 1410 bp in length and encoded a protein of 469 amino acids. The NA showed a high identity at the nucleotide level with A/H5N1 viruses identified in Nigeria (99.06%) and Senegal (99.64%) in 2021 [7]. Interestingly, from the GenBank and GISAID sequences available for the Nigerian and Senegalese A/H5N1 viruses, NAs of two different lengths exist; a full length 469 aa protein and a protein with a 22 aa stalk deletion starting at aa 43. Two of the Nigerian A/H5N1 viruses (GenBank MW961494 and MW961454) possessed a 66 nt deletion in the ORF resulting in a 22 aa stalk deletion (see supplementary Figure S1). None of the A/H5N1 viruses identified in Senegal possessed this deletion. The NA stalk deletions have been identified as a major virulence determinant and it is associated with viral transmission from one host to another especially from wild birds to domestic poultry [9, 10]. Therefore, although the origin of the Lesotho viruses is unknown it is conceivable that due to the presence of a full-length NA, the viruses are recent introductions from wild birds and have not been circulating for extended periods in poultry.

Pairwise analysis of the remaining genome segments [i.e. M, NP, nuclear export protein (NEP), non-structural 1 protein (NS1), polymerase basic protein 1 (PB1) and PB1-F2, polymerase basic protein 2 (PB2), polymerase acid protein (PA) and PA-X] revealed high nucleotide identities (99.12–99.90%) with the Nigerian and Senegalese viruses indicating that A/Chicken/Lesotho/352.3/2021 and A/Chicken/Lesotho/341.10/2021 were not reassortants and highlighting the epidemiological link between the countries.

For PB2, there was a glutamic acid (E) at position 627 revealing that the viruses lacked the mammalian adaptation motif [11]. A P598L mutation in the PB1 was identified which has been shown to enhance polymerase activity in mice and mammalian cells [12]. More recently, Youk et al [13] identified two substitutions in the PB1 of clade 2.3.4.4 viruses which increased polymerase activity in chicken cells. However, these E180D and M317V mutations were not found in the viruses from Lesotho. Likewise, a I109T mutation in the nucleoprotein (NP) which was also shown by Youk et al [13] to be associated with an increased polymerase was not seen in the Lesotho viruses.

The NS1 protein, which varies in length from 202 to 237 in H5NX viruses, was 230 aa in length [14]. It has been recently reported by Blaurock et al [14] that the NS1 proteins in viruses belonging to Clade 2.3.4.4b identified between 2016 and 2020 are frequently 217 aa in length. The authors showed that Clade 2.3.4.4b viruses possessing an NS1 of 217 aa exhibited a number of characteristics that could be deemed beneficial to the virus compared to viruses with a 230 aa NS1. These included a higher capacity for cell-to-cell spread *in vitro*, more efficient blocking of the host interferon-β response and an increased virulence in mice [14].

Although further investigations are required, there is epidemiological evidence that the source of the outbreaks in Lesotho was the supplier in South Africa. Currently, there is not enough data publicly available on the circulation of HPAI A/H5NX in South Africa in 2021 to make meaningful comparisons and to identify a definitive source but there is no doubt that there is a strong molecular epidemiological link between viruses identified in Lesotho and those in western Africa.

Regions of Africa have been recently identified as being at high risk of incursion and co-circulation of HPAI viruses from multiple H5 clades [15]. Western Africa has seen the introduction of A/H5Nx viruses on several occasions since 2005 mainly due to its wetlands which attract large numbers of overwintering migratory birds. Southern Africa is also considered as one of these at-risk regions due, most likely, to intra-African wild bird migrations from western Africa [15]. Indeed, the findings of this study and the identification of HPAIs in southern Africa that are highly related to viruses found also in western Africa would support these previous observations.

In conclusion, this is the first characterization of an HPAI virus from southern Africa in 2021 and has important implications for the management and control of the disease in the region**.** The molecular characterization and analysis of viruses from similar outbreaks in the region (i. e. Botswana and South Africa) will add greatly to our understanding of the movement of these Clade 2.3.4.4b HPAIs within the continent.

## Supplementary Material

Supplemental Material

## References

[1] Lee DH, Bertran K, Kwon JH, et al. Evolution, global spread, and pathogenicity of highly pathogenic avian influenza H5Nx clade 2.3.4.4. J Vet Sci. 2017;18(S1):269–280. 28859267 10.4142/jvs.2017.18.S1.269 PMC5583414

[2] OFFLU. Avian influenza report March 2021 to September 2021. [cited 2022 Jan]. Available at https://www.offlu.org/wp-content/uploads/2021/10/OFFLU-Sept2021-AVIAN-Final.pdf.

[3] WHO. 2018 Protocols influenza virus detection. [cited 2022 Jan]. Available at https://www.who.int/influenza/gisrs_laboratory/Protocols_influenza_virus_detection_Nov_2018.pdf.

[4] Fereidouni SR, Harder TC, Gaidet N, et al. Saving resources: avian influenza surveillance using pooled swab samples and reduced reaction volumes in real-time RT-PCR. J Virol Methods. 2012;186(1-2):119–125. 22925717 10.1016/j.jviromet.2012.08.002

[5] Zhou B, Donnelly ME, Scholes DT, et al. Single-reaction genomic amplification accelerates sequencing and vaccine production for classical and Swine origin human influenza a viruses. J Virol. 2009;83:10309–10313. 19605485 10.1128/JVI.01109-09 PMC2748056

[6] Lee CY, An SH, Kim I, et al. Prerequisites for the acquisition of mammalian pathogenicity by influenza A virus with a prototypic avian PB2 gene. Sci Rep. 2017;7(1):10205. 28860593 10.1038/s41598-017-09560-z PMC5579056

[7] Lo FT, Zecchin B, Diallo AA, et al. Intercontinental spread of Eurasian highly pathogenic avian influenza A(H5N1) to Senegal. Emerg Infect Dis. 2022;28(1):234–237. 34932444 10.3201/eid2801.211401 PMC8714199

[8] Matrosovich M, Stech J, Klenk HD. Influenza receptors, polymerase and host range. Rev Sci Tech. 2009;28(1):203–217. 19618627 10.20506/rst.28.1.1870

[9] Munier S, Larcher T, Cormier-Aline F, et al. A genetically engineered waterfowl influenza virus with a deletion in the stalk of the neuraminidase has increased virulence for chickens. J Virol. 2010;84:940–952. 19889765 10.1128/JVI.01581-09 PMC2798369

[10] Stech O, Veits J, Abdelwhab ES, et al. The neuraminidase stalk deletion serves as major virulence determinant of H5N1 highly pathogenic avian influenza viruses in chicken. Sci Rep. 2015;5:13493. 26306544 10.1038/srep13493 PMC4549673

[11] Herfst S, Schrauwen EJ, Linster M, et al. Airborne transmission of influenza A/H5N1 virus between ferrets. Science. 2012;336(6088):1534–1541. 22723413 10.1126/science.1213362 PMC4810786

[12] Xu C, Hu WB, Xu K, et al. Amino acids 473V and 598P of PB1 from an avian-origin influenza A virus contribute to polymerase activity, especially in mammalian cells. J Gen Virol. 2012;93(3):531–540. 22090209 10.1099/vir.0.036434-0

[13] Youk SS, Leyson CM, Seibert BA, et al. Mutations in PB1, NP, HA, and NA contribute to increased virus fitness of H5N2 highly pathogenic avian influenza virus clade 2.3.4.4 in chickens. J Virol. 2020;95(5):e01675–20. 10.1128/JVI.01675-20 PMC8092828 33268526

[14] Blaurock C, Blohm U, Luttermann C, et al. The C-terminus of non-structural protein 1 (NS1) in H5N8 clade 2.3.4.4 avian influenza virus affects virus fitness in human cells and virulence in mice. Emerg Microbes Infect. 2021;10(1):1760–1776. 34420477 10.1080/22221751.2021.1971568 PMC8432360

[15] Fusaro A, Zecchin B, Vrancken B, et al. Disentangling the role of Africa in the global spread of H5 highly pathogenic avian influenza. Nat Commun. 2019;10(1):5310. 31757953 10.1038/s41467-019-13287-y PMC6874648

