# Supplementary material for: Highly pathogenic avian influenza (A/H5N1) virus outbreaks in Lesotho, May 2021
Source: Emerg Microbes Infect. 2022 Mar 10;11(1):757–60. doi: 10.1080/22221751.2022.2043729 (PMC8920393; doi:10.1080/22221751.2022.2043729)
Supplement: Supplemental Material [file TEMI_A_2043729_SM6464.docx]

**Supplementary material**

**Supplementary Table S1**: Summary of samples tested

| **Description** | **Farm A** | **Farm B** |
| --- | --- | --- |
| Number of nasopharyngeal samples collected | 12 | 13 |
| No of samples tested positive with VetMAX™-Gold AIV RT-qPCR | 12 | 10 |
| No. of samples tested with HA5-specific RT-qPCR | 5 | 5 |
| No. of samples positive with HA5-specific RT-qPCR | 5 | 5 |
| No. of samples sent to APHL for confirmation | 10 | 1 |
| No. of samples confirmed at APHL as H5N1 | 10 | 1 |
| Number of genomes sequenced | 1 | 1 |

**Genome sequencing procedure**

Briefly, 1 µl of each of the primer MBTUni-12-DEG (5’ GCGTGATCAGCRAAAGCAGG 3’) (10 µM) and MBTUni-13 (5’ ACGCGTGATCAGTAGAAACAAGG 3’) (10 µM) was added to 14 µl of total RNA and denatured at 95°C for 2 min and then immediately placed on ice for 2 min. To this primer-RNA mixture the following reagents were added, 25 µl of 2X Reaction Mix (Invitrogen), 8 µl of MgSO_4_, 1 µl of Superscript III Reverse Transcriptase-Platinum Taq High Fidelity (Invitrogen). The thermocycling conditions were reverse transcription at 55°C for 60 min, denaturation at 94°C for 2 min, followed by five cycles of 94°C for 30 sec, 45°C for 30 sec, and 68°C for 4 min and a further 31 cycles of 94°C for 30 sec, 57°C for 30 sec, and 68°C for 4 min with a final elongation at 68°C for 4 min.

The amplified products were checked on a 1.5% (w/v) agarose gel for eight segments ranging from 860 to 2430bp. The amplicons were purified using a 1.8X Agencourt AMPure XP kit (Beckman Coulter, USA). Approximately 50-100ng of the purified amplicons was enzymatically fragmented to 200bp length, using Ion shear Plus reagents (Thermo Fisher Scientific, USA). The fragmented DNA was used to prepare barcoded libraries using an Ion Xpress™ Plus Fragment Library Kit and Ion Xpress barcode adapters (Thermo Fisher Scientific, USA) as per the manufacturer’s protocol, and was size-selected using Pippin Prep (Sage Science, Inc, USA). The pooled barcoded libraries were clonally amplified and enriched using the Ion OneTouch system with the Ion 540TM kit- OT2 reagents and Ion One Touch ES (enrichment system) per the manufacturer’s instructions. Template enriched ISPs with pooled, barcoded, and amplified libraries were loaded onto the Ion540 chip and were sequenced with 500 flows to generate 200 bp reads on the Ion Torrent S5 sequencer (Thermo Fisher Scientific).The raw sequences were cleaned to remove low quality (Phred < 20) and short reads (<50 pb) using fastq-mcf v1.04.676 ([ea-utils](https://github.com/ExpressionAnalysis/ea-utils/tree/wiki)) and the quality of the reads was assessed with FastQC (v. 011.5). De Novo Assemblies were performed using SPAdes (v3.11.1) and CAP3 to produce larger fragments. Using the Denovo assembly's contigs, BLAST searches were performed which identified sequences from an H5N1 isolate from Nigeria (GenBank # MW961452 to MW961455; MW961455 to MW961459 and MW961462) as the most relevant references. After mapping the cleaned raw reads against the reference sequences using BWA (v0.7.17), SAMtools (v1.11) was used to generate Mpileup files and performed variant calling using BCFtools (v1.9), filtering only variant with mapping quality > 20 and minimum coverage depth of 100. The consensus sequences, from reads with mapping quality > 20, were produced with vcfutils.pl (VCFtools v0.1.16) and seqtk (v1.3.106) and compared to the *Denovo* assemblies.

**Supplementary Table S2**: Details of samples for which a full genome was sequenced.

| **Sample #** | **Host** | **Date of collection** | **Sample type** | **Location/GPS** | **Farm ID** |
| --- | --- | --- | --- | --- | --- |
| A/Chicken/Lesotho/341.10/2021 | Chicken | 29/05/2021 | Nasopharyngeal swab | 29°23’49.0” S 27°30’39.5” E | Farm A |
| A/Chicken/Lesotho/352.3/2021 | Chicken | 05/06/2021 | Nasopharyngeal swab | 28**°**55’03.1” S 28**°07’38.8” E** | Farm B |

(a)

114 200

| |

MW961462_A/chicken/Nigeria/VRD21 TCCAAACAGGGAATCATACCAGCCTGAAACATGCAATCAAAGCATCATTACCTATGAGAACAACACCTGGGTAAATCAGACGTATGT

A/Chicken/Lesotho/352.3/2021 TCCAAATAGGGAATCATACCAGCCAGAACCATGCAATCAAAGCATCATTACCTATGAGAACAACACCTGGGTAAATCAGACGTATGT

A/Chicken/Lesotho/341.10/2021 TCCAAATAGGGAATCATACCAGCCAGAACCATGCAATCAAAGCATCATTACCTATGAGAACAACACCTGGGTAAATCAGACGTATGT

MW961494_A/chicken/Nigeria/VRD21 TCCAAACAGGGAATCA------------------------------------------------------------------TATGT

MW961454_A/chicken/Nigeria/VRD21 TCCAAACAGGGAATCA------------------------------------------------------------------TATGT

EPI1866464_A/chicken/Senegal/21V TCCAAACAGGGAATCATACCAGCCTAAACCATGCAATCAAAGCATCATTACCTATGAGAACAACACCTGGGTAAATCAGACGTATGT

EPI1866456_A/chicken/Senegal/21V TCCAAACAGGGAATCATACCAGCCTAAACCATGCAATCAAAGCATCATTACCTATGAGAACAACACCTGGGTAAATCAGACGTATGT

EPI1866448_A/chicken/Senegal/21V TCCAAACAGGGAATCATACCAGCCTAAACCATGCAATCAAAGCATCATTACCTATGAGAACAACACCTGGGTAAATCAGACGTATGT

EPI1866472_A/great-white_pelican TCCAAACAGGGAATCATACCAGCCTGAACCATGCAATCAAAGCATCATTACCTATGAGAACAACACCTGGGTAAATCAGACGTATGT

****** ********* *****

(b)

43 87

| |

MW961462_A/chicken/Nigeria/VRD21 MNPNQRITIIGSICMVIGIVSLMLQIGNIISIWVSHSIQTGNQYQPETCNQSIITYENNTWVNQTYVNISNTNFLAEQAVTSVTLAG

A/Chicken/Lesotho/352.3/2021 MNPNQKIITIGSICMVIGIVSLMLQIGNIISIWVSHSIQIGNQYQPEPCNQSIITYENNTWVNQTYVNISNTNFLAEQAVTSVTLAG

A/Chicken/Lesotho/341.10/2021 MNPNQKIITIGSICMVIGIVSLMLQIGNIISIWVSHSIQIGNQYQPEPCNQSIITYENNTWVNQTYVNISNTNFLAEQAVTSVTLAG

MW961494_A/chicken/Nigeria/VRD21 MNPNQKIITIGSICMVIGIVSLMLQIGNIISIWVSHSIQTGNQ----------------------YVNISNTNFLAEQDVTSVTLAG

MW961454_A/chicken/Nigeria/VRD21 MNPNQKIITIGSICMVIGIVSLMLQIGNIISIWVSHSIQTGNQ----------------------YVNISNTNFLAEQAVTSVTLAG

EPI1866464_A/chicken/Senegal/21V MNPNQKIITIGSICMVIGIVSLMLQIGNIISIWVSHSIQTGNQYQPKPCNQSIITYENNTWVNQTYVNISNTNFLAEQAVTSVTLAG

EPI1866456_A/chicken/Senegal/21V MNPNQKIITIGSICMVIGIVSLMLQIGNIISIWVSHSIQTGNQYQPKPCNQSIITYENNTWVNQTYVNISNTNFLAEQAVTSVTLAG

EPI1866448_A/chicken/Senegal/21V MNPNQKIITIGSICMVIGIVSLMLQIGNIISIWVSHSIQTGNQYQPKPCNQSIITYENNTWVNQTYVNISNTNFLAEQAVTSVTLAG

EPI1866472_A/great-white_pelican MNPNQKIITIGSICMVIGIVSLMLQIGNIISIWVSHSIQTGNQYQPEPCNQSIITYENNTWVNQTYVNISNTNFLAEQAVTSVTLAG

*****.* ****************************** *** ************* ********

**Supplementary Figure S1:** ClustalW alignment of a section of the (a) nucleotide sequence and (b) amino acid sequence of the NA from H5N1 viruses identified in Nigeria, Senegal and Lesotho in 2021
